# Evaluation of choroidal melanin-containing tissue in healthy Japanese subjects by polarization-sensitive optical coherence tomography

**DOI:** 10.1038/s41598-022-07818-9

**Published:** 2022-03-08

**Authors:** Masahiro Miura, Shuichi Makita, Yoshiaki Yasuno, Takuya Iwasaki, Shinnosuke Azuma, Toshihiro Mino, Tatsuo Yamaguchi

**Affiliations:** 1 Department of Ophthalmology, Ibaraki Medical Center, Tokyo Medical University, 3-20-1 Chuo, Ami, Inashiki, Ibaraki 300395 Japan; 2 Computational Optics Group, University of Tsukuba, Tsukuba, Japan; 3 Topcon Corporation, Tokyo, Japan

**Keywords:** Medical imaging, Retina

## Abstract

In this study, the choroidal melanin content in healthy eyes was evaluated with polarization-sensitive optical coherence tomography (PS-OCT). We evaluated 105 healthy eyes of 105 Japanese subjects. The mean thickness of melanin-containing tissue in the choroid (thickness of MeCh) and the choroidal melanin occupancy rate within a 5-mm circular region from the foveal center were calculated using the degree of polarization uniformity obtained by PS-OCT and compared with the choroidal thickness, patient age, and axial length. To evaluate regional variations, the 5-mm circular region was divided into a center area and an outer ring area, and the outer ring area was further divided into four areas (nasal, temporal, superior, and inferior). The mean thickness of MeCh showed a significant positive correlation with the choroidal thickness. The mean choroidal melanin occupancy rate showed a significant positive correlation with age. The mean choroidal melanin occupancy rate of the center area was significantly larger than that of the outer ring area. The mean thickness of MeCh and choroidal melanin occupancy rate of the nasal area were significantly lower than those of other areas. The distribution of melanin-containing tissue in the choroid varies significantly with age and location.

## Introduction

The choroid of the human eye is characterized by an abundance of melanocytes that contain melanin^1^. An important function of choroidal melanin is to maintain the visual performance. Choroidal melanin absorbs stray light to prevent internal reflection of the light from the sclera^1,2^. Choroidal melanin is also an antioxidant that protects the retina from oxidative stress^1–3^. Previous studies have revealed associations of choroidal melanin with the pathogenesis of several chorioretinal diseases, including age-related macular degeneration^4,5^, choroidal melanoma^6^, and Vogt-Koyanagi-Harada disease^7^. Therefore, objective evaluation of choroidal melanin is important to identify its associations with visual functions.

Several studies have focused on quantitative evaluation of the choroidal melanin content in human eyes. In some of these studies, the choroidal melanin occupancy rate was measured using postmortem human eyes^8,9^. However, these studies had several limitations. Because of the difficulty of separating the whole choroid from the retinal pigment epithelium and sclera, only the partial thickness of the choroid or a mixture of the retinal pigment epithelium and choroid could be evaluated^10^. In addition, loss of blood flow in the postmortem eye impedes measurement of the choroidal melanin occupancy rate in the full-thickness choroid. Moreover, evaluation of enucleated human eyes is not an option in clinical practice.

Polarization-sensitive optical coherence tomography (PS-OCT) is a functional extension of OCT in which three-dimensional polarization images of the human eye are acquired in vivo^11^. Melanin in tissues, including the choroid, can scatter light, causing depolarization or polarization scrambling^11–13^. We previously reported that PS-OCT was useful for objective evaluation of choroidal melanin loss in patients with Vogt-Koyanagi-Harada disease^14^. We also evaluated the occupancy rate of melanin-containing tissue in 21 eyes of normal controls^14^. Fujita et al*.*^15^ evaluated the occupancy rate of melanin-containing tissue (average of polarimetric entropy in choroid) of 39 healthy eyes using PS-OCT. These studies compared the occupancy rate of melanin-containing tissue with age and could not confirm a significant relationship^14,15^. Although these preliminary PS-OCT studies provided important information about the choroidal melanin in human eyes in vivo, they had several limitations^14,15^. First, they evaluated only the occupancy rate of melanin-containing tissue, not the thickness of melanin-containing tissue in the full-thickness choroid. Second, because of the relatively small number of subjects, only some aspects of the choroidal melanin-containing tissue in normal eyes could be evaluated.

In the present study, we evaluated the thickness of melanin-containing tissue and its occupancy rate in the choroid using PS-OCT in healthy eyes of a larger number of subjects and investigated their associations with choroidal thickness, patient age, axial length, and location in the macula.

## Methods

### Subjects

This prospective, observational, cross-sectional study was performed using a protocol that adhered to the tenets of the Declaration of Helsinki. Institutional Review Board approval was obtained from Tokyo Medical University (T2019-0072 and T2019-0217). The study was registered in the University Hospital Medical Information Network database (UMIN000039650 and 000039648; http://www.umin.ac.jp/ctr/index-j.htm). The nature of the research and the implications of participating in the study were explained to all potential participants. Written informed consent was obtained from each participant before any study procedures or examinations were performed.

We evaluated 105 healthy eyes of 105 Japanese participants (71 men, 34 women; age range, 22–90 years; mean age, 55.5 years). The right eye of each participant was evaluated. The exclusion criteria were a history or evidence of chorioretinal diseases, vitreoretinal diseases, glaucoma, or a history of any intraocular surgery except cataract surgery. Four eyes underwent phacoemulsification with intraocular lens implantation. Eyes with severe cataracts or other eye diseases that interfered with PS-OCT image quality were excluded from this study. Axial length was measured using an optical biometer (OA-2000; Tomey, Nagoya, Japan). The mean ± standard deviation (SD) axial length was 24.3 ± 1.6 mm (range, 21.7–28.5 mm).

### PS-OCT

**Figure 1:**
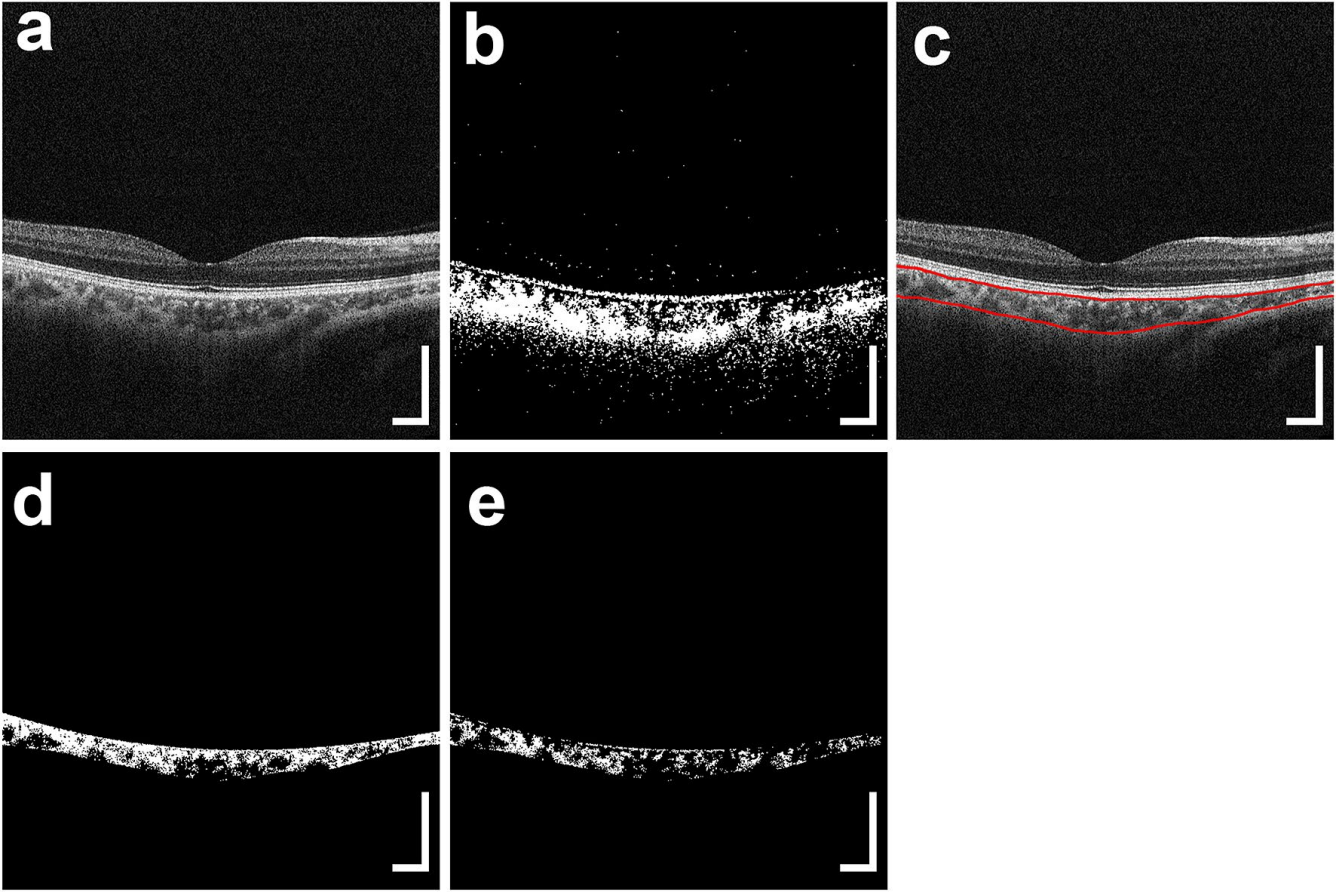
PS-OCT imaging of the right eye of a 48-year-old man. (**a**) Standard OCT B-scan image. (**b**) Binary DOPU B-scan image (DOPU of < 0.8). (**c**) Red lines in the standard OCT B-scan image represent the boundaries for automatic selection of the choroid area. (**d**) Binary B-scan image of choroidal interstitial stroma. (**e**) Binary B-scan image of a low-DOPU area (< 0.8) within the choroidal interstitial stroma. The scale bars represent 500 μm × 500 μm.

A detailed description of the prototype PS-OCT system has been previously reported^16^. The PS-OCT system is a multifunctional swept-source OCT device with 1-μm wavelength band and has a polarization-diversity detection capability. This multifunctional OCT device provides standard OCT, OCT angiography, and degree of polarization uniformity (DOPU) images from a single measurement. The depth resolution was 6 μm in tissue with a refractive index of 1.38. Standard OCT images were obtained by coherent composition of four repeated scans (Fig. 1a)^16^. The DOPU was calculated with Makita’s noise correction using a 3- × 3-pixel kernel (35 μm and 13 μm along the lateral and axial directions, respectively)^17^. The presence of a low DOPU indicated depolarization by multiple scattered lights from melanin. DOPU B-scan images represent areas of low DOPU (< 0.8) in B-scan images (Fig. 1b)^18,19^. A raster scanning protocol with 512 A-lines × 256 B-scans covering a 6.0- × 6.0-mm region of the retina was used for volumetric scans. OCT volumes without significant motion artifacts were used for this study.

**Figure 2:**
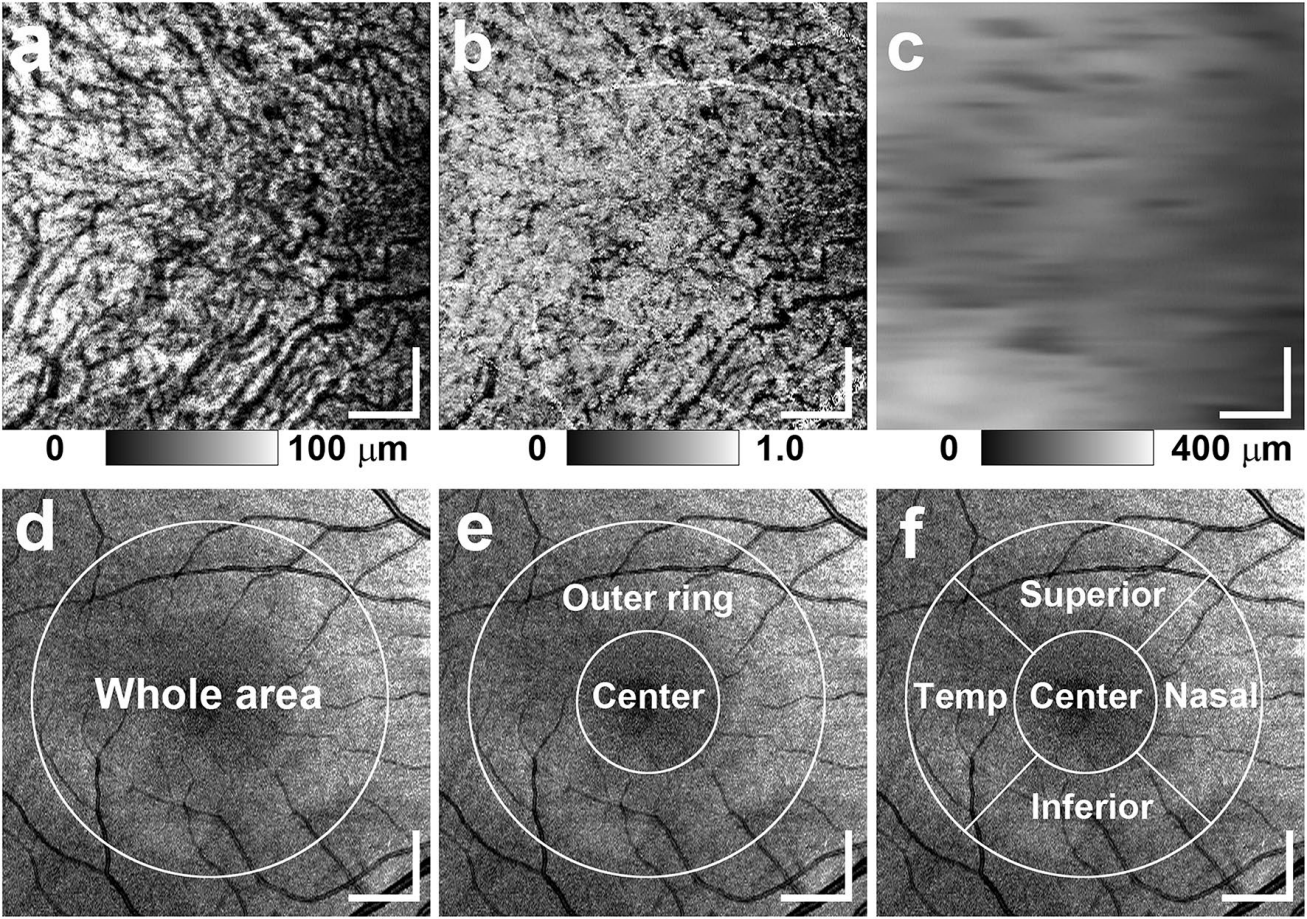
*En-face* PS-OCT images of the eye in Fig. 1. (**a**) Thickness map of melanin-containing tissue in the choroid, (**b**) choroidal melanin occupancy rate map, and (**c**) choroidal thickness map were calculated using volumetric data from PS-OCT. (**d**–**f**) *En-face* projection images of standard OCT. (**d**) The whole area was defined as the inner area of the outer circle. (**e**) The outer ring area was defined as the area between the outer circle and the inner circle, and the center area was defined as the inner area of the inner circle. (**f**) The outer ring area was divided into superior, inferior, temporal (Temp), and nasal areas. The scale bars represent 1.0 mm × 1.0 mm.

For volumetric evaluation of choroidal melanin, we calculated the thickness of the low-DOPU area; i.e., the melanin-rich region in the choroidal interstitial stroma. First, the choroid area in standard OCT B-scan images was automatically selected using an automatic segmentation method identical to the built-in program of a commercially available OCT device (DRI-OCT Triton; Topcon, Tokyo, Japan) (Fig. 1c). Second, the choroid areas in standard OCT B-scan images were binarized using the local Otsu method followed by median filtering using Fiji image processing software^20^ to separate the choroidal area into vessel and interstitial areas (Fig. 1d). Third, the area of low DOPU (< 0.8), defined as the total area of low DOPU within the choroidal interstitial stroma, was calculated from the B-scan DOPU images (Fig. 1e). This threshold value of low DOPU (< 0.8) was determined according to our previous PS-OCT study on choroidal melanin-containing tissue^14^. The thickness of melanin-containing tissue in the choroid (thickness of MeCh) was calculated by counting the number of pixels of low DOPU (< 0.8) within the choroidal interstitial stroma in each A-line. The thickness of MeCh represents the amount of choroidal melanin-containing tissue in the full-thickness choroid. The choroidal melanin occupancy rate was calculated from the percentage area of low DOPU (< 0.8), defined as the occupancy of the low-DOPU area within the choroidal interstitial stroma. The choroidal melanin occupancy rate represents the density of melanin-containing tissue in the interstitial stroma. Using volumetric data from PS-OCT, a thickness map of MeCh and a choroidal melanin occupancy rate map were generated (Fig. 2a, b). The choroidal thickness map was calculated according to the automatically selected choroid area (Fig. 2c). The choroidal thickness map represents the thickness of the whole choroid, including both vessels and interstitial stroma. In contrast, both the thickness of MeCh and the choroidal melanin occupancy rate represent the distribution of melanin-containing tissue in the interstitial stroma excluding vessels.

The volumetric scan was divided by inner and outer circles centered on the fovea with diameters of 2 and 5 mm, respectively. The mean thickness of MeCh, mean choroidal melanin occupancy rate, and mean choroidal thickness of the whole area were calculated for the inner area of the outer circle (Fig. 2d). For regional evaluation of the thickness of MeCh. choroidal melanin occupancy rate, and choroidal thickness, we defined the “center area” as the area within the center circle and the “outer ring area” as the area between the inner and outer circles. We compared the center area with the outer ring area (Fig. 2e). In addition, the outer ring area was evenly split into four areas; i.e., the superior, inferior, nasal, and temporal areas. In consequence, we compared five sectors consisting of the center area and these four areas (Fig. 2f). For quantitative measurements, transverse magnification of PS-OCT images was calibrated using a modification of Littman’s method^21^.

To assess reproducibility, we evaluated the choroidal thickness, thickness of MeCh, and choroidal melanin occupancy rate of the whole area in 12 eyes (9 men, 3 women; age range, 28–63 years; mean age, 43.2 years). The coefficient of variation was calculated using four repeated measurements for each eye.

Statistical analyses were performed with IBM SPSS Statistics for Windows, version 27.0 (IBM Corp., Armonk, NY, USA). Statistical significance was defined as P < 0.05.

## Results

The mean ± SD choroidal thickness, thickness of MeCh, and choroidal melanin occupancy rate of the whole area of all 105 eyes were 204.9 ± 79.3 µm (range, 61.3–482.4 µm), 33.2 ± 13.4 µm (range, 10.8–70.4 µm), and 0.38 ± 0.12 (range, 0.12–0.73), respectively.

**Figure 3:**
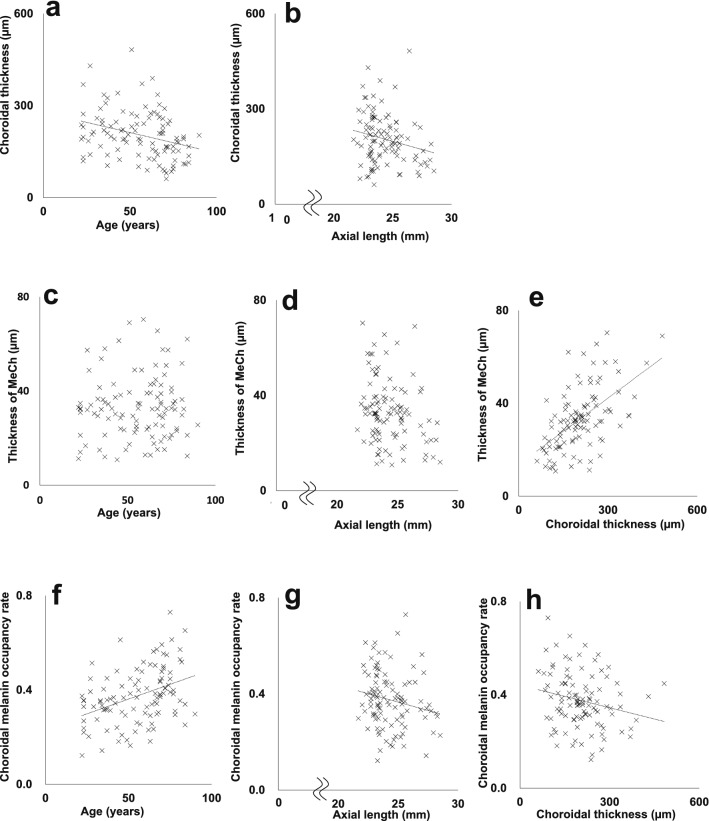
Scatterplots of choroidal thickness as a function of (**a**) age and (**b**) axial length. Scatterplots of thickness of melanin-containing tissue in the choroid (thickness of MeCh) as a function of (**c**) age, (**d**) axial length, and (**e**) choroidal thickness. Scatterplots of choroidal melanin occupancy rate as a function of (**f**) age, (**g**) axial length, and (**h**) choroidal thickness. Dotted lines indicated the regression lines of relationships with significant correlations.

The mean choroidal thickness of the whole area was compared with age and axial length by simple linear regression analysis. The choroidal thickness showed a significant negative correlation with both age and axial length (Table 1; Fig. 3). The mean thickness of MeCh and choroidal melanin occupancy rate of the whole area were compared with age, axial length, and choroidal thickness by simple linear regression analysis. The thickness of MeCh showed a significant positive correlation with choroidal thickness (Table 1; Fig. 3). The choroidal melanin occupancy rate showed a significant positive correlation with age and a significant negative correlation with both axial length and choroidal thickness (Table 1; Fig. 3).

**Table 1:** Summary of simple linear regression analysis of choroidal thickness, thickness of MeCh, and choroidal melanin occupancy rate.

		Correlation coefficient	P value
Choroidal thickness	Age	− 0.308	0.01*
Choroidal thickness	Axial length	− 0.210	0.031*
Thickness of MeCh	Age	0.060	0.54
Thickness of MeCh	Axial length	− 0.260	0.07
Thickness of MeCh	Choroidal thickness	0.565	< 0.001*
Choroidal melanin occupancy rate	Age	0.392	< 0.001*
Choroidal melanin occupancy rate	Axial length	− 0.192	0.049*
Choroidal melanin occupancy rate	Choroidal thickness	− 0.218	0.025*

Thickness of MeCh: thickness of melanin-containing tissue in the choroid.

*Statistically significant correlation.

Subsequently, a multiple linear regression analysis was conducted to consider interactions among parameters with significant correlations. Stepwise multiple linear regression analysis of the choroidal thickness with two parameters (age and axial length) revealed that the choroidal thickness showed a significant negative correlation with both age and axial length (Table 2). Stepwise multiple linear regression analysis of the choroidal melanin occupancy rate with three parameters (age, axial length, and choroidal thickness) revealed that the choroidal melanin occupancy rate showed a significant positive correlation only with age (Table 2). In this step, the thickness of MeCh showed a significant correlation only with the choroidal thickness in the simple linear regression analysis, and it was removed from the multiple linear regression analysis.

**Table 2:** Summary of stepwise multiple linear regression analysis of choroidal thickness and choroidal melanin occupancy rate.

Parameters	β	95% CI	P value
**Choroidal thickness**			
Age	− 0.474	− 0.663 to − 0.286	< 0.001*
Axial length	− 0.405	− 0.592 to − 0.217	< 0.001*
**Choroidal melanin occupancy rate**			
Age	0.308	0.093 to 0.523	0.007*
Axial length	− 0.096	− 0.304 to 0.111	0.38
Choroidal thickness	− 0.114	− 0.315 to 0.086	0.17

*CI* confidence interval.

*Statistically significant correlation.

The mean ± SD coefficients of variation of four repeated measurements were 0.072 ± 0.040, 0.066 ± 0.041, and 0.026 ± 0.034 for the thickness of MeCh, choroidal melanin occupancy rate, and choroidal thickness of the whole area, respectively.

Next, we evaluated the regional differences in the choroidal thickness, thickness of MeCh, and choroidal melanin occupancy rate (Table 3). First, we compared the center area and outer ring area. The mean choroidal thickness and choroidal melanin occupancy rate of the center area were significantly larger than those of the outer ring area (P < 0.001 and P = 0.005, respectively; Wilcoxon signed rank test). The mean thickness of MeCh of the center area showed no significant difference with that of the outer ring area (P = 0.66; Wilcoxon signed rank test). Second, we compared the areas divided into five sectors: the center, superior, inferior, nasal, and temporal areas. The mean choroidal thickness, thickness of MeCh, and choroidal melanin occupancy rate of the nasal area were significantly smaller than those of the other four areas (P < 0.001, P < 0.001, and P < 0.001, respectively; Friedman and Bonferroni post-hoc test). The mean thickness of MeCh of the temporal area was significantly larger than that of the center, superior area, and inferior area (P < 0.001, P = 0.006, and P < 0.001, respectively; Friedman and Bonferroni post-hoc test). The mean choroidal melanin occupancy rate of the temporal area was significantly larger than that of the inferior area (P = 0.03; Friedman and Bonferroni post-hoc test).

**Table 3:** Summary of choroidal thickness, thickness of MeCh, and choroidal melanin occupancy rate at each sector.

Area	Choroidal thickness (μm)	Thickness of MeCh (μm)	Choroidal melanin occupancy rate
Center	218.3 ± 85.6 (68.7–534.9)	33.4 ± 13.7 (8.8–72.8)	0.39 ± 0.14 (0.11–0.81)
Superior	216.4 ± 79.6 (57.4–485.2)	33.9 ± 13.1 (7.2–67.8)	0.38 ± 0.12 (0.07–0.76)
Inferior	207.3 ± 84.8 (58.4–469.0)	31.9 ± 14.5 (8.5–81.0)	0.37 ± 0.12 (0.15–0.78)
Nasal	173.8 ± 83.2 (58.8–516.6)	28.8 ± 15.3 (6.4–80.0)	0.33 ± 0.12 (0.08–0.74)
Temporal	212.0 ± 83.0 (65.2–473.4)	37.9 ± 15.0 (11.2–73.2)	0.40 ± 0.13 (0.13–0.78)
Outer ring	202.4 ± 78.6 (60.0–472.6)	33.1 ± 13.5 (10.6–71.1)	0.37 ± 0.12 (0.12–0.71)

Data are presented as mean ± standard deviation (range).

Thickness of MeCh: thickness of melanin-containing tissue in the choroid, Center: center area, Superior: superior area, Inferior: inferior area, Nasal: nasal area, Temporal: temporal area, Outer ring: outer ring area.

## Discussion

Despite the importance of melanin-containing tissue in the choroid in the pathogenesis of chorioretinal diseases, there are no established methods in clinical practice to objectively evaluate melanin-containing tissue in the choroid in vivo*.* In the present study, quantitative measurements of melanin-containing tissue in healthy eyes were performed in vivo using PS-OCT. The amount of melanin-containing tissue varied substantially with age, axial length, and location. The thickness of MeCh increased as the choroidal thickness increased. The choroidal melanin occupancy rate increased with advancing age. The choroidal melanin occupancy rate of the center area was significantly larger than that of the outer ring area. Both the thickness of MeCh and choroidal melanin occupancy rate of the nasal area were significantly smaller than those of other areas. We also evaluated the choroidal thickness in this study. The choroidal thickness showed a significant negative correlation with age and axial length. The choroid was thicker in the center area than in the outer ring area. Among the five sectors, the choroid was thinner in the nasal area. These findings regarding the choroidal thickness coincide with previous reports^22–25^.

In the present study, the thickness of MeCh showed a significant positive correlation with the choroidal thickness; however, the choroidal melanin occupancy rate showed no significant correlation with the choroidal thickness. This finding indicates that the choroidal melanin occupancy rate is independent of the choroidal thickness. Therefore, the amount of melanin-containing tissue should increase with increasing choroidal thickness because the choroidal melanin occupancy rate showed no significant correlation with the choroidal thickness. Choroidal melanin is highly efficient at absorbing and scattering incident visible light^26,27^. Choroidal melanin should induce a negative effect on the visibility of deep structures in color fundus images by the attenuation of incident light. The visibility of choroidal vessels might deteriorate as melanin-containing tissues increase. A previous study showed a negative correlation between the choroidal thickness and visibility of choroidal vessels^28^. The thickness of melanin-containing tissues might be an important contributing factor to the visibility of choroidal vessels.

In the present study, the choroidal melanin occupancy rate showed a positive correlation with age. Weiter et al.^8^ evaluated 38 donor eyes with optical measurements and could not confirm a significant correlation between the choroidal melanin occupancy rate and age. Nevertheless, they reported that the choroidal melanin content tended to decrease with aging, despite a lack of specific data in their paper^8^. Fujita et al*.*^15^ evaluated 39 healthy eyes with PS-OCT and could not confirm a correlation between age and the occupancy rate of melanin-containing tissue (average of polarimetric entropy in choroid). Our previous PS-OCT study involving 21 healthy eyes also could not confirm a correlation between age and the choroidal melanin occupancy rate^14^. The present study was conducted with 105 healthy eyes, and this larger number of examined eyes might have led to our statistically significant findings with respect to age. Previous ex vivo and in vivo studies showed that human ocular melanocytes retain the capacity to produce melanin pigment throughout adult life^29,30^. A study involving cell cultures showed that melanin significantly increased in senescent uveal melanocytes^29^. The results of the present study reaffirmed the increase in choroidal melanin with aging. However, there is a counterargument for the increase in choroidal melanin with aging. A transmission electron microscopy study of aged human choroidal melanocytes showed emptiness in the cytoplasm caused by the loss of melanosomes^31^. Additional histological studies will be required to resolve this controversy about the aging-related changes in choroidal melanin.

In the present study, the choroidal melanin occupancy rate of the center area was significantly larger than that of the outer ring area. Weiter et al*.*^8^ evaluated the choroidal melanin occupancy rate using eyes obtained from autopsies and reported that the choroidal melanin content increased from the periphery to the posterior pole. Our results are consistent with their study. In addition, the present study showed that both the thickness of MeCh and choroidal melanin occupancy rate of the nasal area were significantly smaller than those of other areas. Possible regional variation of choroidal melanin should be considered in further investigations of the pathophysiology of choroidal melanin.

This study has several limitations. First, this study only evaluated Japanese patients. Racial differences in choroidal melanin have been reported^8^. Investigation of non-Japanese patients is required to further evaluate these racial differences. In this study, the choroidal parameters (thickness of MeCh, choroidal melanin occupancy rate, and choroidal thickness) showed large variations. For accurate evaluation of the large variation in these parameters, a larger number of subjects is required to establish a normal database. Second, although a previous study showed a monotonic relationship between the DOPU and melanin, measurement of melanin-containing tissue could be affected by the DOPU kernel size or melanin packing density in choroidal melanocytes. Therefore, thickness maps of MeCh do not represent the actual thickness of choroidal melanin, although they are proportional to the thickness of melanin-containing tissue. In addition, we used the threshold value of low DOPU (< 0.8) according to our previous PS-OCT study on choroidal melanin^14^. Further study is required to determine the appropriate threshold value for choroidal melanin measurement. Third, a previous PS-OCT study showed a decreased DOPU in hard exudate^32^. The possible presence of exudative tissue should be considered for the further clinical application of our method to various chorioretinal diseases. Fourth, choroidal melanin is a complex macromolecule consisting of pheomelanin and eumelanin^2,33^. Investigating the role of these high-order structures of choroidal melanin is beyond the capability of our PS-OCT measurement. Fifth, there are currently no alternative techniques to measure the human melanin-containing tissue in the choroid in vivo. Without a reference standard, we cannot fully validate our results. Sixth, in this study, the volumetric scan was divided into five sectors by inner and outer circles with diameters of 2 and 5 mm. Volumetric analysis of melanin-containing tissue was influenced by measurement fields. In a future study, comparison with different sector analyses might be helpful for improvement of our method. Seventh, the physical mechanism of depolarization has not yet been fully proven. Another possible mechanism is the scattering by nonspherical particles^34^. In this study, we assessed the metrics derived from the DOPU to investigate choroidal melanin-containing tissue; i.e., the thickness of MeCh and the choroidal melanin occupancy rate. Many factors affect the DOPU, such as the wavelength band used for OCT imaging^35^, and shape and size of the spatial kernel to compute the DOPU^36,37^. These resulted in the variation of the metrics used in this study. Special attention would be required to compare individual eyes measured by different PS-OCT systems or conditions.

Another important limitation is the state of polarization of incident light. DOPU measurement is known to highly depend on the incident polarization state^38–40^. Our PS-OCT system used single-mode optical fibers in the sample arm^16^, and the polarization state of the incident beam to the eye was not fully stable and may have varied among measurements. In contrast, previous clinical studies of the DOPU, mostly performed at the Medical University of Vienna^18,19,36^, used a circularly polarized incident beam, and the variation in the DOPU dependent on the incident polarization state would be minimal^39^; i.e., only the variations of corneal and neural retinal birefringence among patients should be considered. This suggests two additional limitations of this study. First, our DOPU value may have unpredictably varied among patients and/or measurements. Second, the DOPU values shown in this study, as well as the DOPU values obtained with our single-mode fiber-based PS-OCT^16^ in general, could not be directly compared with those in studies that used different PS-OCT systems^18,19,36^. Careful consideration is required to quantify and generalize our results. However, the results of this study showed that metrics derived from the binarized DOPU with our PS-OCT system have the potential to facilitate evaluation of the choroidal melanin distribution in human eyes. Further study is required to develop robust regulation of the states of polarization for practical use of PS-OCT systems.

In conclusion, this study showed the usefulness of PS-OCT to evaluate the melanin-containing tissue in the choroid. DOPU measurement with PS-OCT can be used to noninvasively evaluate the thickness of the melanin-containing tissue and occupancy rate. PS-OCT has the potential to evaluate the association of the melanin-containing tissue in the choroid with various chorioretinal diseases. PS-OCT is a promising tool to evaluate the clinical significance of the melanin-containing tissue in the choroid.

## Supplementary Information

Supplementary Table S1.
