# Supplementary material for: Evaluation of choroidal melanin-containing tissue in healthy Japanese subjects by polarization-sensitive optical coherence tomography
Source: Sci Rep. 2022 Mar 8;12:4048. doi: 10.1038/s41598-022-07818-9 (PMC8904585; doi:10.1038/s41598-022-07818-9)
Supplement: Supplementary file 1 — Supplementary Table S1. [file 41598_2022_7818_MOESM1_ESM.pdf]

Evaluation of choroidal melanin-containing tissue in healthy Japanese subjects by polarization-sensitive optical coherence tomography

Masahiro Miura, Shuichi Makita, Yoshiaki Yasuno, Takuya Iwasaki, Shinnosuke Azuma, Toshihiro Mino, Tatsuo Yamaguchi

Supplementary Table S1.

Summary of characteristics of subjects, choroidal thickness of the whole area, thickness of melanin-containing tissue in the choroid of the whole area, and choroidal melanin occupancy rate of the whole area.

Subject	Age (years)	Axial length (mm)	Choroidal thickness (μm)	Thickness of melanin-containing tissue in the choroid (μm)	Choroidal melanin occupancy rate
1	56	24.5	176.2	34.8	0.441
2	84	23.5	136.0	21.2	0.340
3	82	23.2	109.2	32.3	0.519
4	72	22.9	240.1	50.9	0.407
5	78	23.1	180.8	24.1	0.295
6	47	24.1	191.6	29.9	0.367
7	66	22.7	335.9	44.9	0.348
8	76	22.3	121.2	34.6	0.614
9	71	23.4	61.3	15.1	0.500
10	68	25.4	208.4	29.7	0.305
11	69	25.6	92.3	18.6	0.439
12	75	23.2	103.4	21.3	0.391
13	53	23.5	218.6	29.4	0.319
14	61	22.2	259.6	39.1	0.328
15	48	24.9	180.2	23.5	0.293
16	81	22.0	191.7	36.4	0.354
17	59	22.1	296.6	70.4	0.519
18	84	23.7	114.2	12.4	0.252
19	72	23.1	164.7	32.5	0.408
20	71	22.2	80.1	20.6	0.492
21	77	22.3	183.3	32.2	0.398

22	66	23.3	139.6	25.7	0.385
23	71	24.4	166.8	46.9	0.554
24	56	24.9	188.7	32.6	0.399
25	69	24.0	219.6	38.9	0.435
26	65	27.0	152.3	43.0	0.563
27	69	24.8	209.8	34.4	0.381
28	81	23.3	110.3	29.8	0.529
29	27	22.9	429.9	57.4	0.393
30	63	25.5	176.5	12.8	0.182
31	63	23.1	157.8	39.2	0.508
32	54	24.8	239.4	22.4	0.223
33	51	26.4	482.4	69.0	0.448
34	58	23.2	241.2	48.9	0.478
35	71	25.3	164.1	30.5	0.386
36	84	25.0	166.6	62.1	0.652
37	70	22.9	87.2	25.2	0.592
38	75	25.6	93.4	41.3	0.730
39	57	22.5	371.2	34.6	0.255
40	55	26.9	139.0	27.3	0.369
41	54	23.3	265.8	49.0	0.423
42	75	23.2	167.0	39.6	0.474
43	67	23.4	303.5	37.4	0.342
44	49	24.4	205.7	15.0	0.188
45	64	23.3	278.7	32.3	0.256
46	70	22.5	290.6	57.6	0.478
47	60	27.3	89.5	12.8	0.302
48	41	25.6	191.2	32.6	0.369
49	34	26.9	252.5	41.5	0.334
50	46	28.3	189.2	28.5	0.307
51	45	23.1	218.1	61.4	0.613
52	26	24.0	184.8	16.8	0.203
53	51	23.8	231.6	39.4	0.385
54	63	23.9	389.1	42.8	0.291
55	42	24.8	246.7	42.9	0.344
56	43	23.4	340.8	30.3	0.241
57	37	25.3	210.0	35.1	0.357

58	37	28.5	104.5	12.0	0.227
59	46	24.5	222.0	38.3	0.416
60	31	25.3	258.9	30.5	0.275
61	43	23.9	156.4	37.3	0.481
62	36	22.9	308.2	36.4	0.317
63	28	26.3	206.3	48.8	0.514
64	35	22.6	335.4	53.7	0.404
65	39	23.5	199.4	34.5	0.362
66	35	23.1	219.5	32.2	0.315
67	61	23.5	127.2	23.7	0.407
68	33	23.3	290.5	36.6	0.350
69	37	24.1	324.9	58.0	0.450
70	51	23.5	273.4	17.4	0.165
71	67	22.7	209.9	32.4	0.366
72	23	27.9	119.5	14.0	0.238
73	77	25.0	193.9	35.7	0.383
74	62	23.4	274.5	24.7	0.231
75	23	25.2	369.1	34.8	0.220
76	70	23.9	151.3	28.7	0.450
77	74	22.8	84.9	19.8	0.420
78	44	24.5	123.7	10.8	0.182
79	69	22.8	233.7	35.3	0.380
80	65	25.4	151.9	17.7	0.234
81	90	21.7	203.2	25.6	0.298
82	67	23.4	103.4	15.2	0.298
83	23	23.3	197.4	30.9	0.356
84	29	25.8	214.1	34.1	0.329
85	39	25.3	232.8	28.3	0.313
86	34	27.3	241.6	14.9	0.143
87	78	23.0	152.4	23.1	0.311
88	22	23.3	237.5	11.3	0.122
89	23	24.4	272.5	32.2	0.269
90	64	27.4	124.4	20.5	0.340
91	33	27.6	167.5	29.4	0.355
92	67	23.9	261.7	65.6	0.572
93	69	25.3	257.7	42.6	0.371

94	73	22.8	250.8	57.4	0.549
95	66	23.3	272.8	51.0	0.504
96	81	23.4	198.5	51.8	0.572
97	55	27.1	100.9	23.7	0.488
98	37	28.2	138.9	21.3	0.319
99	23	27.8	131.3	21.2	0.329
100	22	25.2	190.7	33.0	0.375
101	23	24.3	230.1	32.6	0.354
102	27	25.9	140.5	32.0	0.448
103	75	23.1	150.9	32.7	0.447
104	46	24.6	281.2	24.0	0.209
105	55	25.6	191.6	25.0	0.261
